# Predictive Low-Glucose Suspend Necessitates Less Carbohydrate Supplementation to Rescue Hypoglycemia: Need to Revisit Current Hypoglycemia Treatment Guidelines

**DOI:** 10.1089/dia.2020.0619

**Published:** 2021-06-29

**Authors:** Jordan E. Pinsker, Amy Bartee, Michelle Katz, Amy Lalonde, Richard Jones, Eyal Dassau, Howard Wolpert

**Affiliations:** ^1^ Sansum Diabetes Research Institute, Santa Barbara, California, USA.; ^2^ Eli Lilly and Company, Indianapolis, Indiana, USA.; ^3^ Eli Lilly and Company, Cambridge, Massachusetts, USA.

**Keywords:** Carbohydrate, hypoglycemia, Predictive low-glucose suspend, Type 1 diabetes

## Abstract

Current guidelines recommend 15–20 g of carbohydrate (CHO) for treatment of mild to moderate hypoglycemia. However, these guidelines do not account for reduced insulin during suspensions with predictive low-glucose suspend (PLGS). We assessed insulin suspensions, hypoglycemic events, and CHO treatment during a 20-h inpatient evaluation of an investigational system with a PLGS feature, including an overnight basal up-titration period to activate the PLGS. Among 10 adults with type 1 diabetes, there were 59 suspensions; 7 suspensions were associated with rescue CHO and 5 with hypoglycemia. Rescue treatment consisted of median 9 g CHO (range: 5–16 g), with no events requiring repeat CHO. No rescue CHO were given during or after insulin suspension for the overnight basal up-titration. To minimize rebound hyperglycemia and needless calorie intake from hypoglycemia overtreatment, updated guidance for PLGS systems should reflect possible need to reduce CHO amounts for hypoglycemia rescue associated with an insulin suspension. The clinical trial was registered with ClinicalTrials.gov (NCT03890003).

## Introduction

Predictive low-glucose suspend (PLGS)^1–4^ and hybrid closed-loop systems^5,6^ are now commercially available and increasingly being used in type 1 diabetes (T1D) management. PLGS systems automatically suspend or decrease insulin delivery in response to impending hypoglycemia. When these systems are not able to prevent a hypoglycemic episode entirely, this diminution in delivered insulin may decrease the amount of carbohydrates (CHOs) required to treat hypoglycemia and the needless consumption of additional calories. In addition, overtreatment of hypoglycemia can lead to rebound hyperglycemia and contribute to increased glycemic variability and diminished time in range.^7^ However, clinical guidelines for CHO treatment of hypoglycemia^8,9^ have not been revised to reflect the impact of reduced insulin delivered by advanced insulin delivery systems.

The objective of this analysis was to evaluate insulin suspension events and related hypoglycemia treatment and glycemic responses during the use of an investigational PLGS insulin management system.

## Methods

### Participants and study design

Data were derived from an inpatient single site study (NCT03890003). For this study, subjects (*N* = 10) were adults aged ≥18 years to <66 years with a clinical diagnosis of T1D for ≥2 years and using an insulin pump for ≥6 months. Additional inclusion criteria were a hemoglobin A1C (HbA1c) of ≥6.0 to ≤9.0 and body mass index (BMI) of 18.5–37 kg/m^2^. Relevant exclusion criteria include severe hypertension (>160/100) or unstable coronary artery disease, adrenal insufficiency, renal insufficiency [glomerular filtration rate <45 mL/(min ·1.73 m^2^)] and severe hypoglycemia or diabetic ketoacidosis within the past 6 months. Participants provided informed consent before any study procedures and the protocol was approved by the Advarra IRB and conducted according to the International Conference on Harmonization, Good Clinical Practice guidelines, and the Declaration of Helsinki. The study was conducted under an investigational device exemption from the U.S. Food and Drug Administration.

The study consisted of a screening visit, 7-day lead in period with pump setting optimization, and a 20-h inpatient period at the Sansum Diabetes Research Institute (Santa Barbara, CA) with a brief follow-up phone visit 1–2 days later. On the first day of the inpatient period, subjects were placed on an investigational pump filled with Humalog^®^ (insulin lispro) and embedded with an investigational PLGS algorithm and the Dexcom G6 continuous glucose monitoring (CGM; Dexcom, San Diego, CA). Pump settings from the lead in period were adjusted if needed as determined by the study team and entered into a handheld controller (locked down Android smartphone). Settings were adjusted so that PLGS would occur if glucose values were predicted to be <85 mg/dL in 30 min to counteract potential CGM bias.

Subjects were treated with CHOs based upon a confirmatory blood glucose <60 mg/dL with symptoms of hypoglycemia or <54 mg/dL regardless of symptoms, or at the discretion of the study investigator. The amount of CHO (food, juice, or glucose tabs) for hypoglycemia treatment was determined by the study investigator (J.E.P.).

### Basal titration challenge

Subjects were admitted to the clinical study site before dinner and consumed a dinner that consisted of a low-fat meal with no more than 15 g saturated fat. The basal up-titration challenge was started after subjects' glucose was <180 mg/dL and ∼4 h after the start of dinner. Basal insulin doses were titrated upward by 0%–25% every 90 min based upon the current and previous 30 min glucose values to gradually lower blood glucose and trigger the PLGS system.^10,11^ Once insulin was suspended by the PLGS system, basal up-titration terminated. Capillary blood glucose and ketones were monitored every 30 min for 2 h after resumption of basal insulin; the last set of readings during this period marked the end of the titration challenge period for analysis. The following morning subjects were discharged home after breakfast.

For these analyses, the postprandial period was defined as the time between the start of the meal until (1) 4 h after the meal, (2) the start of the basal titration challenge, or (3) the time the PLGS system was removed before discharge, whichever occurred first. The overnight period was defined as the start of the basal titration challenge, around 10 pm–6 am. The evening meal was provided at 17:30 ± 15 min. The glucose resulting from a suspension was determined by the change in glucose from the minimum value after the suspension until the inflection point. The inflection point was defined as the point at which the CGM glucose reached a slope ≤0 based on at least three consecutive CGM readings. CHO averted was calculated as the amount of insulin that was not delivered based on the programmed basal rate for the duration of the suspension multiplied by the CHO to insulin ratio.

## Results

### Participant characteristics

Participants (*n* = 10), 40% male, 100% white, 30% Hispanic or Latino, were aged (mean ± standard deviation) 39.0 ± 13.0 years with a duration of T1D of 23.2 ± 8.1 years and BMI of 27.1 ± 3.9 kg/m^2^. Diabetes management characteristics included a mean HbA1c of 7.2% ± 0.6 and mean insulin dose of 0.6 ± 0.2 U/(kg·day).

### Insulin suspensions

There were 52 PLGS-triggered insulin suspensions across all 10 subjects where no CHO supplementation was required (Table 1). Most events occurred in the overnight period (*n* = 26) or in the postprandial period (*n* = 16), with 10 events occurring neither in the periods defined as postprandial nor overnight. None of these suspensions were associated with hypoglycemia. These suspensions were 41 ± 37 min in duration and, among those suspensions lasting for at least 20 min with an observable inflection point (*n* = 35), resulted in a modest glucose increase of 22.6 ± 24.5 mg/dL. The suspended insulin translated into 7.1 ± 6.6 g of CHO averted. No rescue CHO were given during or after insulin suspension for the overnight basal up-titration.

**Table 1.** Summary of Suspensions in the Postprandial and Overnight Periods According to Carbohydrate Administration

	PLGS suspensions: CHO administered	PLGS suspensions: CHO NOT administered		
Study period	Postprandial^a^	Postprandial^a^	Overnight^b^	Overall^c^
Suspensions, *n*	7	16	26	52
Subjects experiencing ≥1 susp., *n* (%)	5 (50)	9 (90)	9 (90)	10 (100)
Susp. duration (HH:MM)	1:10 (0:37)	0:32 (0:36)	0:57 (0:39)	0:41 (0:37)
CGM glucose ROC at susp. [mg/(dL·min)]	−1.4 (0.8)	−1.5 (1.1)	−0.5 (0.3)	−0.9 (0.8)
Min. CGM glucose during susp. (mg/dL)	65.9 (15.5)	108.0 (26.6)	88.2 (11.3)	94.8 (19.0)
Susp. associated with hypoglycemia, *n*	5	0	0	0
CHO administered (g)	9.9 (4.8)	—	—	—
CHO averted (g)^d^	14.2 (10.1)	5.6 (6.2)	9.4 (7.2)	7.1 (6.6)
Susp. lasting >2 PLGS control cycles, *n*	7	12	23	41
Susp. lasting >2 PLGS control cycles with observed glucose inflection, *n*	6	8	23	35
Time to glucose inflection (HH:MM)	1:20 (0:34)	0:36 (0:51)	0:52 (0:41)	0:49 (0:43)
Glucose at inflection (mg/dL)	120.8 (28.9)	103.4 (20.2)	109.7 (24.9)	110.3 (24.8)
Resulting increase in glucose (mg/dL)^e^	54.5 (32.6)	13.4 (14.5)	23.3 (25.5)	22.6 (24.5)

Summaries are mean (SD) unless otherwise stated.

There are some suspensions that were neither postprandial nor overnight.

^a^ Time from the start of the meal until 4 h later, the start of the basal titration challenge, or the time the AID system is removed before discharge, whichever occurs first.

^b^ 10 pm–6 am.

^c^ The entire time subjects were on the PLGS system.

^d^ The amount of insulin not delivered multiplied by the subject's ICR.

^e^ Difference in CGM glucose at the inflection point and at the minimum.

CGM, continuous glucose monitoring; CHO, carbohydrate; ICR, insulin to CHO ratio; PLGS, predictive low glucose suspend; ROC, rate of change; SD, standard deviation.

**FIG. 1.**
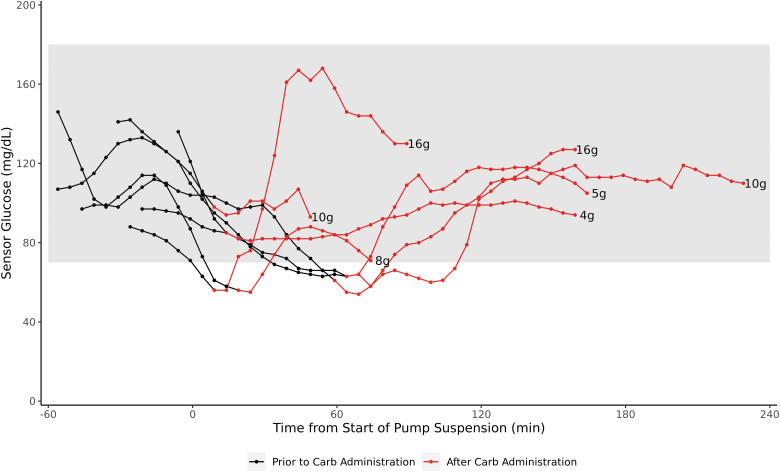
Individual sensor glucose trajectories before, during, and after insulin pump suspension during which CHO were administered. Black values are before CHO administration and red values are after CHO administration with the grams of CHO administered noted with each trajectory. Subjects' glucose levels were observed to remain in range (70–180 mg/dL) after judicious CHO administration. CHO, carbohydrate.

There were seven PLGS-triggered insulin suspensions in which CHO were administered by site staff, and all occurred in the postprandial period. Five of these suspensions were associated with hypoglycemia. Subjects received an average of 9.9 ± 4.8 g of CHO, range 5–16 g and 14.2 ± 10.1 g CHO were averted during this time through the insulin suspension. These suspensions were for a duration of 70 ± 37 min. These episodes were associated with an increase in glucose of 54.5 ± 32.6 mg/dL, range 30–105 mg/dL resulting in a glucose at inflection of between 88 and 168 mg/dL (Fig. 1, Supplementary Table S1).

There were two suspensions in which 16 g of CHO were administered with a resulting increase in glucose of 75 and 105 mg/dL.

## Conclusions

This study demonstrated that hypoglycemia occurring during use of PLGS may require less CHO treatment than currently recommended by clinical guidelines.^9,12^ This is illustrated by the limited number of instances of CHO used to treat hypoglycemia and by calculating the CHO averted based upon the decrement in programmed basal insulin preceding these episodes. Avoiding overtreatment of hypoglycemia may avoid subsequent hyperglycemia and increased glycemic variability while preventing unnecessary caloric intake from CHO.

The American Diabetes Association^12^ and Diabetes Canada^9^ recommend 15–20 g of glucose or CHO for the treatment of hypoglycemia. These recommendations come from early research showing the glycemic response to varied forms and amounts of CHO, before the advent of CGM to provide rate of change information and algorithms to modulate insulin doses.^13–15^ A recent review describes how the evidence base for these treatment amounts was supported by very limited quality evidence.^16^ There are little available data for hypoglycemia treatment amounts during the use of systems that proactively limit insulin delivery to avoid or lessen hypoglycemia. Multiple studies of automated insulin delivery systems continue to dictate treatment of hypoglycemia with 15–20 g of CHO per protocol^17–20^ despite increasing recognition that this may lead to hyperglycemia from clinical experience.^21,22^ Notably, the pivotal study protocol that led to FDA approval of the Control-IQ system recommended CHO treatment for hypoglycemia but did not specify a CHO treatment amount.^6^

Recognition that suspended insulin delivery may modify the amount of CHO needed is increasing, although clinical evidence continues to be limited. The potential for overzealous CHO treatment to interfere with the function of PLGS systems has been described in an earlier study of PLGS technology, aptly entitled “Let the Algorithm do the Work.”^7^ Open APS documentation discusses the risk for hyperglycemia if users continue to treat hypoglycemia with 15 g of CHO, and Loop similarly acknowledges that less insulin on board ahead of hypoglycemia will often lead to fewer grams of CHO needed for treatment.^23,24^ In addition, the CARES paradigm recommends that persons with diabetes treat with fewer grams of CHO if their hypoglycemia has been preceded by an insulin suspension.^22^

Insulin suspension preceding the hypoglycemia is just one of a series of factors that should be evaluated when determining how to treat mild to moderate hypoglycemia events. The type of sugar and food used to treat the hypoglycemia can also have a profound effect on the resulting glycemic excursions.^13,14,25,26^ In pediatrics, a more individualized weight-based treatment is advised given the wide range of body weight in children.^9,27^ The International Society for Pediatric and Adolescent Diabetes guidelines further note that the precise amount of CHO should also take into account the type of insulin therapy, proximity of exercise, and whether the type of CHO administered is sucrose, fructose, or glucose.^27^ Adults, too, might benefit from considering those factors in addition to the duration of suspension and any insulin on board from a preceding bolus.

This study has some limitations. Although being a highly controlled inpatient study allowed detailed calculation of the insulin delivered, CHO averted, and the amount of CHO given, results may not be fully generalizable to an outpatient setting. Furthermore, this study was not designed as a test of reduced CHO treatment in response to hypoglycemia while on a PLGS system, and thus the episodes are limited, and the amount of CHO given was dependent on investigator discretion. However, the methods of this study were sufficient for the intent of this report, which was to raise the consciousness in the clinical community of the potential need for more limited CHO administration to treat hypoglycemia with the use of more advanced insulin delivery systems.

Careful research, clinician experience, and guideline changes may, by themselves, be insufficient to avoid overtreatment of hypoglycemia when preceded by insulin suspension. Patients may continue to overtreat hypoglycemia secondary to fear of hypoglycemia, desire to limit the unpleasant sensations caused by hypoglycemia, or CGM lag time.^28^ Thus, clinicians will need to provide explicit and likely repeated retraining on the amount of CHO needed when hypoglycemia is preceded by insulin suspension.

Hypoglycemia episodes may often be treatable with less CHO than clinical guidelines recommend during PLGS and closed-loop insulin delivery, where insulin suspension precedes hypoglycemia. However, it is difficult for most persons with diabetes to fully integrate the complex interplay between the duration of the suspension, remaining insulin on board, and glucose rate of change, as well as any exercise-related factors, to estimate the CHO amount required for treatment of hypoglycemia. Differences between PLGS systems such as with the predicted glucose suspension threshold might further add complexity to determining precise CHO requirements across systems. OpenAps already has an app to guide CHO dose adjustment in the setting of hypoglycemia for users of their system.^23^ This situation-specific guidance on CHO treatment is a valuable tool and should be considered for incorporation into commercially available insulin delivery systems. Finally, careful studies, such as those first done to establish the guideline of 15–20 g of CHO, should be conducted to inform CHO treatment of hypoglycemia when preceded by an insulin suspension in the era of modern insulin delivery systems.

## Supplementary Material

Supplemental data

## References

[1] Forlenza GP, Li Z, Buckingham BA, et al.: Predictive low-glucose suspend reduces hypoglycemia in adults, adolescents, and children with type 1 diabetes in an at-home randomized crossover study: results of the PROLOG trial. Diabetes Care 2018;41:2155–2161 30089663 10.2337/dc18-0771

[2] Pinsker JE, Leas S, Müller L, Habif S: Real world improvements in hypoglycemia in an insulin-dependent cohort with diabetes mellitus pre/post tandem basal-IQ technology remote software update. Endocr Pract 2020;26:715–721 10.4158/EP-2019-0554 33471639

[3] Zhong A, Choudhary P, McMahon C, et al.: Effectiveness of automated insulin management features of the MiniMed((R)) 640G sensor-augmented insulin pump. Diabetes Technol Ther 2016;18:657–663 27672710 10.1089/dia.2016.0216 PMC5111481

[4] Pinsker JE, Dassau E: Predictive low-glucose suspend to prevent hypoglycemia. Diabetes Technol Ther 2017;19:271–276 28426238 10.1089/dia.2017.0064

[5] Bergenstal RM, Garg S, Weinzimer SA, et al.: Safety of a hybrid closed-loop insulin delivery system in patients with type 1 diabetes. JAMA 2016;316:1407–1408 27629148 10.1001/jama.2016.11708

[6] Brown SA, Kovatchev BP, Raghinaru D, et al.: Six-month randomized, multicenter trial of closed-loop control in type 1 diabetes. N Engl J Med 2019;381:1707–1717 31618560 10.1056/NEJMoa1907863 PMC7076915

[7] Biester T, Kordonouri O, Holder M, et al.: “Let the Algorithm Do the Work”: reduction of hypoglycemia using sensor-augmented pump therapy with predictive insulin suspension (SmartGuard) in pediatric type 1 diabetes patients. Diabetes Technol Ther 2017;19:173–182 28099035 10.1089/dia.2016.0349 PMC5359639

[8] American Diabetes Association: Introduction: Standards of Medical Care in Diabetes—2020. Diabetes Care 2020;43(Supplement 1):S1–S231862741

[9] Yale JF, Paty B, Senior PA: Hypoglycemia. Can J Diabetes 2018;42 Suppl 1:S104–S108 29650081 10.1016/j.jcjd.2017.10.010

[10] Buckingham B, Chase HP, Dassau E, et al.: Prevention of nocturnal hypoglycemia using predictive alarm algorithms and insulin pump suspension. Diabetes Care 2010;33:1013–1017 20200307 10.2337/dc09-2303 PMC2858164

[11] Buckingham BA, Bailey TS, Christiansen M, et al.: Evaluation of a predictive low-glucose management system in-clinic. Diabetes Technol Ther 2017;19:288–292 28221823 10.1089/dia.2016.0319

[12] American Diabetes Association: 6. Glycemic Targets: Standards of Medical Care in Diabetes-2019. Diabetes Care 2019;42(Suppl 1):S61–S70 30559232 10.2337/dc19-S006

[13] Brodows RG, Williams C, Amatruda JM: Treatment of insulin reactions in diabetics. JAMA 1984;252:3378–3381 6389915

[14] Slama G, Traynard PY, Desplanque N, et al.: The search for an optimized treatment of hypoglycemia. Carbohydrates in tablets, solutin, or gel for the correction of insulin reactions. Arch Intern Med 1990;150:589–593 2310277

[15] Wiethop BV, Cryer PE: Alanine and terbutaline in treatment of hypoglycemia in IDDM. Diabetes Care 1993;16:1131–1136 8375243 10.2337/diacare.16.8.1131

[16] Villani M, de Courten B, Zoungas S: Emergency treatment of hypoglycaemia: a guideline and evidence review. Diabet Med 2017;34:1205–1211 28477413 10.1111/dme.13379

[17] DeBoer MD, Cherñavvsky DR, Topchyan K, et al.: Heart rate informed artificial pancreas system enhances glycemic control during exercise in adolescents with T1D. Pediatr Diabetes 2017;18:540–546 27734563 10.1111/pedi.12454

[18] Forlenza GP, Ekhlaspour L, Breton M, et al.: Successful at-home use of the tandem control-IQ artificial pancreas system in young children during a randomized controlled trial. Diabetes Technol Ther 2019;21:159–169 30888835 10.1089/dia.2019.0011 PMC6909715

[19] Huyett LM, Ly TT, Forlenza GP, et al.: Outpatient closed-loop control with unannounced moderate exercise in adolescents using zone model predictive control. Diabetes Technol Ther 2017;19:331–339 28459617 10.1089/dia.2016.0399 PMC5510043

[20] Ly TT, Roy A, Grosman B, et al.: Day and night closed-loop control using the integrated medtronic hybrid closed-loop system in type 1 diabetes at diabetes camp. Diabetes Care 2015;38:1205–1211 26049550 10.2337/dc14-3073

[21] Boughton CK, Hartnell S, Allen JM, et al.: Training and support for hybrid closed-loop therapy. J Diabetes Sci Technol 2020 doi:10.1177/1932296820955168 PMC8739578 32914648

[22] Messer LH, Berget C, Forlenza GP: A clinical guide to advanced diabetes devices and closed-loop systems using the CARES paradigm. Diabetes Technol Ther 2019;21:462–469 31140878 10.1089/dia.2019.0105 PMC6653788

[23] Open APS: Optimizing Settings and Making Changes. https://openaps.readthedocs.io/en/latest/docs/Resources/clinician-guide-to-OpenAPS.html#optimizing-settings-and-making-changes (accessed 1026, 2020)

[24] Loop Tips: Low Treatments. https://kdisimone.github.io/looptips/how-to/low-treat/ (accessed 1026, 2020)

[25] Erbas IM, Abaci A, Anik A, et al.: Comparison of the effectiveness of simple carbohydrates on hypoglycemic episodes in children and adolescents with type 1 diabetes mellitus: a randomized study in a diabetes camp. Pediatr Diabetes. 2020;21:1249–1255 32662200 10.1111/pedi.13077

[26] McTavish L, Wiltshire E: Effective treatment of hypoglycemia in children with type 1 diabetes: a randomized controlled clinical trial. Pediatr Diabetes 2011;12(4 Pt 2):381–387 21443586 10.1111/j.1399-5448.2010.00725.x

[27] Clarke W, Jones T, Rewers A, et al.: Assessment and management of hypoglycemia in children and adolescents with diabetes. Pediatr Diabetes 2009;10(Suppl 12):134–145 19754624 10.1111/j.1399-5448.2009.00583.x

[28] Basu A, Dube S, Slama M, et al.: Time lag of glucose from intravascular to interstitial compartment in humans. Diabetes 2013;62:4083–4087 24009261 10.2337/db13-1132 PMC3837059

