# Supplementary material for: Predictive Low-Glucose Suspend Necessitates Less Carbohydrate Supplementation to Rescue Hypoglycemia: Need to Revisit Current Hypoglycemia Treatment Guidelines
Source: Diabetes Technol Ther. 2021 Jun 29;23(7):512–6. doi: 10.1089/dia.2020.0619 (PMC8252907; doi:10.1089/dia.2020.0619)
Supplement: Supplemental data [file Supp_TableS1.pdf]

Supplemental Table.

Comparing the response to PLGS with and without carbohydrate supplementation

Summary *a	Postprandial *b		Overnight *c	Overall *d
	Carbs *e	No Carbs	No Carbs	No Carbs
n (%)	5 (50.0)	9 (90.0)	9 (90.0)	10 (100.0)
Number of Suspensions	7	16	26	52
Suspensions per subject	0.7 (0.8)	1.6 (1.0)	2.6 (1.6)	5.2 (2.1)
Number of Suspensions Associated with a Hypoglycemic event	5	0	0	0
Number of Suspensions Associated with a Rebound Hyperglycemic Event	0	0	0	0
Number of Suspensions Associated with a Rebound Hypoglycemic Event	1	3	0	3
Suspension Duration (HH:MM:SS)	1:10:00 (0:37:12)	0:31:34 (0:35:37)	0:56:38 (0:38:39)	0:41:29 (0:37:09)
Time since last meal (HH:MM:SS)	0:25:00	0:12:30 (0:02:53)		
CGM Glucose at Suspension (mg/dL)	100.0 (17.6)	117.9 (24.7)	97.0 (10.1)	102.8 (18.5)
ROC at Suspension (mg/dL/min)	-1.4 (0.8)	-1.5 (1.1)	-0.5 (0.3)	-0.9 (0.8)

*a - All summaries are the mean (SD) except for 'n (%)' and 'Number of suspensions'.

*b - The time period defined by the start of the meal until 4 hours later or the start of the next challenge.

*c - The time period defined by the start of the basal titration challenge around 10pm on Day 1 until 6am on Day 2.

*d - The entire time the patients are on the AID system.

*e - Carbohydrates were only administered in the postprandial period.

Note: 5 suspensions during which carbs were not administered within the postprandial period did not demonstrate a glucose inflection point following the start of the resumption and prior to subsequent carbohydrate consumption (snack or rescue) or suspension or discharge

n = number of subjects with a suspension during the study period; CGM = Continuous Glucose Monitoring; PLGS = Predictive Low Glucose Suspend; ROC = Rate of Change
